# Malaria diagnostic capacity in health facilities in Ethiopia

**DOI:** 10.1186/1475-2875-13-292

**Published:** 2014-07-29

**Authors:** Tesfay Abreha, Bereket Alemayehu, Yehualashet Tadesse, Sintayehu Gebresillassie, Abebe Tadesse, Leykun Demeke, Fanuel Zewde, Meseret Habtamu, Mekonnen Tadesse, Damtew Yadeta, Dawit Teshome, Addis Mekasha, Kedir Gobena, Henock Bogale, Zenebe Melaku, Richard Reithinger, Hiwot Teka

**Affiliations:** 1 ICAP at Columbia University’s Mailman School of Public Health, Addis Ababa, Ethiopia; 2 ICAP at Columbia University’s Mailman School of Public Health, New York, USA; 3 Oromia Regional Health Bureau, Addis Ababa, Ethiopia; 4 Dire Dawa Regional Health Bureau, Dire Dawa, Ethiopia; 5 RTI International, Washington DC, USA; 6 USAID Ethiopia, Addis Ababa, Ethiopia

## Abstract

**Background:**

Accurate early diagnosis and prompt treatment is one of the key strategies to control and prevent malaria in Ethiopia where both *Plasmodium falciparum* and *Plasmodium vivax* are sympatric and require different treatment regimens. Microscopy is the standard for malaria diagnosis at the health centres and hospitals whereas rapid diagnostic tests are used at community-level health posts. The current study was designed to assess malaria microscopy capacity of health facilities in Oromia Regional State and Dire Dawa Administrative City, Ethiopia.

**Methods:**

A descriptive cross-sectional study was conducted from February to April 2011 in 122 health facilities, where health professionals were interviewed using a pre-tested, standardized assessment tool and facilities’ laboratory practices were assessed by direct observation.

**Results:**

Of the 122 assessed facilities, 104 (85%) were health centres and 18 (15%) were hospitals. Out of 94 health facilities reportedly performing blood films, only 34 (36%) used both thin and thick smears for malaria diagnosis. The quality of stained slides was graded in 66 health facilities as excellent, good and poor quality in 11(17%), 31 (47%) and 24 (36%) respectively. Quality assurance guidelines and malaria microscopy standard operating procedures were found in only 13 (11%) facilities and 12 (10%) had involved in external quality assessment activities, and 32 (26%) had supportive supervision within six months of the survey. Only seven (6%) facilities reported at least one staff’s participation in malaria microscopy refresher training during the previous 12 months. Although most facilities, 96 (79%), had binocular microscopes, only eight (7%) had the necessary reagents and supplies to perform malaria microscopy. Treatment guidelines for malaria were available in only 38 (31%) of the surveyed facilities. Febrile patients with negative malaria laboratory test results were managed with artemether-lumefantrine or chloroquine in 51% (53/104) of assessed health facilities.

**Conclusions:**

The current study indicated that most of the health facilities had basic infrastructure and equipment to perform malaria laboratory diagnosis but with significant gaps in continuous laboratory supplies and reagents, and lack of training and supportive supervision. Overcoming these gaps will be critical to ensure that malaria laboratory diagnosis is of high-quality for better patient management.

## Background

Globally, an estimated of 3.4 billion people live in areas at risk of malaria. In 2012 there were 207 million cases of malaria and 627, 000 deaths; 90% of these deaths occurred in sub-Saharan Africa [1]. Approximately 75% of Ethiopia’s landmass is endemic for malaria transmission, with 58 million people at risk of infection and disease [2]. Malaria is one of the top ten causes of morbidity, accounting for 17% of all cases and 8% of health facility admissions in 2012 [3].

Accurate early diagnosis and prompt treatment of malaria is among the core strategies to prevent and control malaria [4]. Despite significant funding for HIV/AIDS, tuberculosis and malaria in the past decade (e. g. through the Global Fund to Fight AIDS, Tuberculosis and Malaria; the U.S. President’s Emergency Plan for AIDS Relief and President’s Malaria Initiative), laboratory systems in developing countries remain weak. Major challenges include poor physical infrastructure and inadequate supplies of materials and reagents, limited human capacity, lack of policies and strategic plans, and limited synergies between clinical and laboratory services [5,6]. Strong laboratory systems not only ensure that curative interventions are more effective (e. g., by avoiding prescription of artemisinin combination therapy (ACT) to patients with non-malarial causes of fever), but also affect treatment-seeking behaviour (i. e. health facilities with a strong laboratory service tend to experience greater access by patients than facilities with a weak laboratory service) [7].

Following the WHO recommendations of universal diagnostic testing for all suspected malaria cases [8], Ethiopia scaled up diagnostic testing for malaria at all levels of the public sector’s health service delivery system: multispecies rapid diagnostic tests (RDTs) are used at community-level health posts and malaria microscopy is carried out at district-level health centres as well as district-, zonal- and regional-level hospitals [9].

Microscopy requires a functional laboratory set-up with quality diagnostic services and trained laboratory personnel [5]. Published peer-reviewed literature on in-country laboratory capacity, including for malaria, is scarce. Existing data comes almost exclusively from grey literature [10-13], showing low malaria microscopic diagnostic capacity that ranges from 25% in Uganda, to 33% in Tanzania and 37% in Rwanda. In 2009, assessment of malaria diagnosis capacity in 69 health facilities in Oromia Regional State showed that although most facilities (i. e. 51 (88%)) did provide malaria microscopy services, they faced a myriad of challenges, including limitations in trained personnel, functional laboratory equipment and microscopes, standard operating procedures (SOP) and guideline availability, and continuous supply of necessary reagents and materials [14].

The objective of the survey presented here was to expand the assessment of malaria diagnostic capacity beyond the five administrative zones of Oromia covered in 2009 to facilities within all of Oromia’s 18 zones, as well as Dire Dawa City Administration.

## Methods

### Study design and site selection

A descriptive cross-sectional study was conducted from February to April 2011. A convenience sampling method was used to select 126 health facilities, from 108 malaria-endemic districts in 18 administrative zones of Oromia National Regional State and Dire Dawa City Administration. Oromia is the largest regional state in Ethiopia covering 359,620 km^2^ (latitude 8°00’ N, longitude 39° 00’ E); the topography is diverse, with altitude ranging from 500 m to 4,377 m above sea level (asl). The annual mean temperature is 19°C (range: 10 - 30°C); annual rainfall ranges from 400 – 2,400 mm, with bi-modal rainy seasons. Dire Dawa is a chartered city located in the eastern part of Ethiopia (latitude 9° 36’ N, longitude 41° 52’ E). It covers an area of 1213 km^2^; with altitude ranging from 960 m to 2,450 m asl. The annual mean temperature is 25°C (range: 18 - 31°C); average annual rainfall is 604 mm. Between July 2012 and June 2013, an estimated 1,058,240 malaria cases were reported in Oromia and 661 cases in Dire Dawa, accounting 23% and 0.01% of the country’s malaria burden, respectively [15].

### Data collection and analysis

Three assessment teams, composed of staff from the Oromia Regional Health Bureau (ORHB), Oromia Regional Reference Laboratory, zonal and district health offices and Columbia University’s International Center for AIDS Care and Treatment Programs (ICAP) in Ethiopia, carried out the survey, collecting all data using a pre-tested, structured data collection tool. The tool has a questionnaire section that included: (i) general information about the facilities, including access to water and power supply; (ii) information on type of malaria laboratory services provided, including availability of human resources, essential laboratory and clinical equipment and supplies, quality assurance (QA) protocols for malaria laboratory diagnostics, status of biosafety and implementation; and, (iii) information on laboratory-confirmed malaria data at each health facility. In addition the tool included check list for other observed variables. The survey approach included interviews of health facility personnel, review of secondary data of laboratory registers and direct observation of services provided.

The collected data were checked for completeness and consistency, and double entered into a SPSS17.0 (SPSS Inc. Chicago, USA) database. Descriptive statistical analysis was done using STATA 11.0 (STATA Corp. Texas, USA).

### Ethical consideration

The survey was not reviewed by an ethical review committee. As it was part of the routine programme management, permission for conducting the survey was obtained from ORHB and Dire Dawa City Administration Health Bureau. No patient information or identifiers were collected in the survey; only data on facilities’ characteristics were collected, with a summary report provided to relevant authorities.

## Results

### Facility characteristics and infrastructure

**Figure 1:**
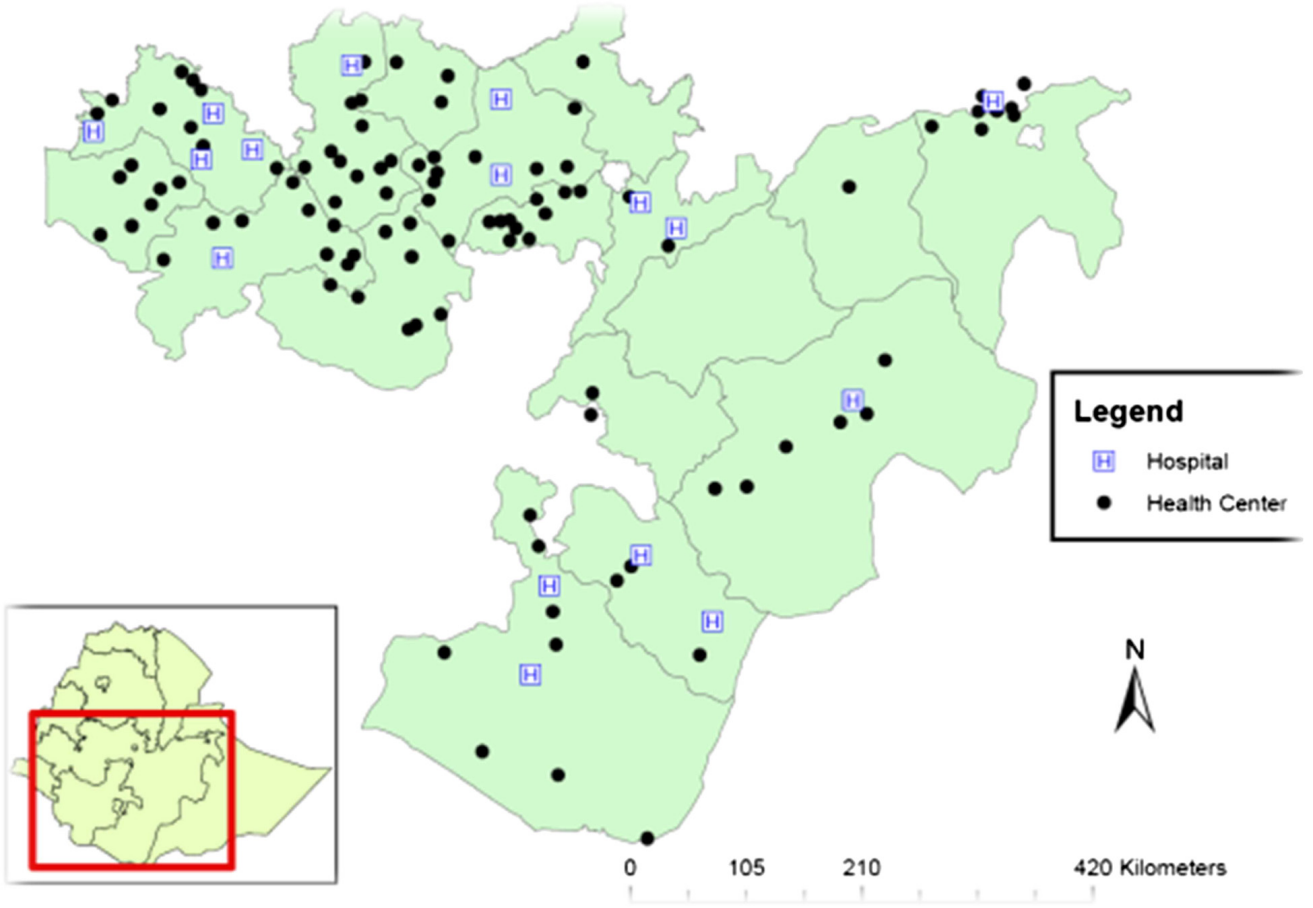
Location of surveyed facilities.

Among the 126 selected facilities, no data were available from four facilities due to the absence of a laboratory unit or laboratory personnel at the time of the survey. Of the 122 surveyed facilities, 104 were health centres and 18 were hospitals (Figure 1). The median numbers of beds per health centre were five (range: 0–40) and 75 (range: 28–200) in hospitals. All but one facility were public sector facilities.

Although 97 (80%) facilities had access to electricity, 63 (65%) reported frequent power interruptions, with 55 (87%) reporting power interruptions at least once per week; 49 (40%) facilities had back-up generators for electricity. Of the assessed laboratories, 86 (70%) had access to water; 76 (88%) of these were using tap water as their source, with the remainder using well or rain water. Facilities reporting interruption of water and electric power services affecting the diagnosis of malaria were 38 (31%) and 69 (57%), respectively.

### Laboratory equipment and consumables

Of surveyed facilities, 119 (98%) had at least one functional binocular microscope, 102 (84%) and 66 (54%) had functional electric and manual centrifuges, respectively. Slide staining racks were available in 30 (25%) facilities, and slide drying racks and staining troughs were available in 29 (24%) and 21 (17%) facilities, respectively. No facility had a functional pH meter. Other major and minor laboratory equipment are listed in Tables 1 and 2. Only one facility reported to have laboratory staff trained and designated to maintain equipment.

**Table 1:** Major laboratory equipment in 122 facilities surveyed

**Equipment**	**Number of facilities with functional equipment**	**Number of facilities with non-functional equipment**	**Number of facilities without equipment**
Binocular microscope	119 (98%)	53 (43%)	1 (0.8%)
Spare bulbs for microscopes	35 (29%)	No data	83 (68%)
Electric centrifuges	102 (84%)	26 (21%)	16 (13%)
Manual centrifuges	66 (54%)	16 (13%)	51 (42%)
Weighing scale	22 (18%)	3 (2%)	95 (78%)
Colorimeter	14 (11%)	3 (2%)	105 (86%)
Haemoglobinometer	66 (54%)	5 (4%)	51 (42%)
Micro HCT centrifuge	44 (36%)	8 (6.5%)	73 (60%)
Refrigerator	66 (54%)	10 (8%)	50 (41%)
Autoclave	19 (16%)	2 (1.6%)	99 (81%)
Blood cell analyzer	15 (12%)	2 (1.6%)	100 (82%)
pH meter	0 (0%)	1 (0.8%)	115 (94%)

**Table 2:** Minor laboratory equipment in the 122 facilities surveyed

**Equipment**	**Number of facilities with functional equipment**	**Number of facilities without equipment**
Slide staining racks	30 (25%)	88 (72%)
Slide drying racks	29 (24%)	88 (72%)
Automatic pipettes	46 (38%)	72 (59%)
Timers	94 (77%)	23 (19%)
Tally counters	21 (17%)	97 (76%)
Differential counters	82 (67%)	36 (30%)
Fridge thermometers	34 (28%)	84 (69%)
Staining troughs	21 (17%)	97 (80%)
Slide storage boxes	65 (53%)	53 (43%)
Beakers	43 (35%)	74 (61%)
Flasks	44 (36%)	72 (59%)

Problems in continuous supply of laboratory consumables was reported from 55/117 (47%) surveyed facilities for Giemsa stain solution, 36 (30%) for microscopic slides, 90/118 (76%) for lens tissue, 45/118 (38%) for immersion oil, and 80/114 (70%) for lens cleaning solution (Table 3). Malaria RDTs were available in 70 (57%) facilities.

**Table 3:** Supply of laboratory consumables in the 122 facilities surveyed

**Laboratory consumables**	**Facilities with problem of supplies**	**Facilities without problem of supplies**	**Facilities which do not use specified consumable**
Giemsa stain	55 (47%)	56 (48%)	6 (5%)
Field stain	15 (15%)	4 (4%)	83 (81%)
Lancets	30 (26%)	77 (68%)	7 (6%)
Pasteur pipettes	60 (52%)	46 (40%)	9 (8%)
Sharps containers	37 (32%)	78 (68%)	0 (0%)
Microscope slides	36 (30%)	78 (64%)	0 (0%)
Immersion oil	45 (38%)	72 (62%)	0 (0%)
Lens tissue	90 (76%)	25 (21%)	3 (3%)
Lens cleaning solution	80 (70%)	19 (17%)	15 (13%)
Biohazard container	75 (66%)	39 (34%)	0 (0%)
Alcohol	43 (36%)	75 (64%)	0 (0%)
Bleach	42 (35.5%)	75 (63.5%)	1 (1%)
Gloves	56 (48%)	60 (51%)	1 (1%)
First aid kit	56 (62%)	8 (9%)	53 (45%)
Glycerol	58 (49%)	31 (26%)	29 (25%)
Methanol AR grade	47 (40%)	55 (46.5%)	16 (13.5%)
Na_2_HPO_4_ buffer	65 (55%)	9 (8%)	44 (37%)
KH_2_OPO_4_ buffer	66 (56%)	7 (6%)	45 (38%)
pH paper	84 (71%)	7 (6%)	27 (23%)
Filter paper	86 (73%)	23 (20%)	8 (7%)

In all the surveyed facilities the reagents were made available through the respective district or zonal health offices. Reagents were stored at room temperature with the exception of one facility, where reagents were stored in a refrigerator. Correctly labelled reagents were found in 74 (61%) facilities, although only 19 (16%) showed a clear record of expiry dates.

The minimum package of equipment and reagent required for malaria microscopy (i. e., microscope, slide, staining vessel, lens cleaning tissue, measuring cylinder, optical lens cleaner, immersion oil, Giemsa stain, methanol, gloves, blood lancets, sanitary cotton and ethanol) was available in eight (7%) of the health facilities.

### Human resources and training

Hospitals had a greater number of skilled professional staff, probably due to serving a larger population: all medical specialists 51 (100%) and 95 (92%) of general practitioners reported in the 122 facilities worked in hospitals. In total, 275 laboratory personnel were found to work in the surveyed health facilities, with 120 (44%) working in hospitals and 155 (56%) in health centres (Table 4). The number of laboratory staff in each health facilities ranged from one to 21 (health centres: one to six; hospital: three to 21); the majority (i. e., 74%) of surveyed facilities had two to three laboratory staff and 44 (36%) had only one.

**Table 4:** Distribution of clinical and laboratory health workforce in 122 facilities surveyed

**Profession**	**Number working in hospitals (%)**	**Number working in health centers (%)**
Clinical		
Medical specialists (all type)	51 (60%)	34 (40%)
General practitioners	95 (92%)	8 (8%)
Health officer	64 (33%)	131 (67%)
Nurse	737 (46%)	882 (54%)
Health assistant	12 (38%)	20 (62%)
Laboratory staff	120 (44%)	155 (56%)

The qualification of the laboratory personnel varied from diploma level in medical laboratory science to those with postgraduate studies. Of surveyed facilities with laboratory personnel, 89 (73%) had at least one laboratory technician with diploma level undergraduate training, 80 (66%) had laboratory technologists with BSc degree level undergraduate training; staff with a postgraduate degree were found in five laboratories.

In seven (6%) of the surveyed facilities, at least one staff reported having participated in malaria microscopy refresher training within the previous year. Other refresher training, such as training on RDTs was reported in three (2%) facilities, and laboratory personnel trained in supply chain management were found in eight (7%) facilities.

### Biosafety

During the survey, laboratory staff were observed to wear protective gloves and coats in 83 (68%) and 91 (75%) of facilities, respectively. Among the surveyed facilities, hand washing areas were observed in 65 (53%), and 27 (22%) had properly labelled and collected biohazardous materials. Sharp boxes were used to dispose contaminated sharps in 91 (75%) facilities, with remaining facilities using biohazard bags and waste bins (Table 5).

**Table 5:** Status of biosafety operation

**Description of biosafety operation**	**Yes, observed**	**Available but do not use**	**Not available**
People in laboratory wear protective gloves	83 (68%)	12 (10%)	22 (18%)
People in laboratory wear protective coats	91 (75%)	8 (7%)	16 (13%)
Hand washing facility in laboratory	65 (53%)	4 (3%)	46 (38%)
Disinfectants/decontaminants use	81 (66%)	3 (2%)	30 (25%)
Antiseptic use	86 (70%)	3 (2%)	24 (20%)

### Treatment guidelines, laboratory request and related forms

Malaria treatment guidelines were available in 38 (31%) of surveyed facilities. Laboratory request forms were available in 51 (42%) facilities and those without request forms used plain paper; only 37 (30%) facilities had properly completed request forms and seven (6%) facilities did not use request forms despite their availability. Request forms included the clinician’s clinical impressions in 13 (11%) of the facilities assessed. Registers were found in 118 (97%) facilities, the majority of them (i. e., 93/118 (79%)) keeping neat and legible records. Other records, including records for laboratory equipment maintenance, were available in 11 (9%) facilities.

### Laboratory services for malaria

Although, theoretically, all facilities should provide microscopy services, only 94 (77%) facilities performed blood films. In these facilities, a total of 220,341 blood films had been performed within the year preceding the survey, i. e., an annual average of 2,344 blood films per facility. RDTs were also observed and reported to be used in 38 (31%) and 27 (22%) of the facilities, respectively. Of the surveyed facilities, 34 (28%) prepared both thick and thin blood films for malaria diagnosis; 57 (47%) prepared thick films only and three (2%) prepared thin films only. Malaria species identification was performed in 78 (64%) facilities; three (2%) facilities reported or were observed to perform parasite counts. Haemoglobin estimation was done in 78 (64%) facilities, out of which 31 (40%) used the haematocrit technique, 29 (37%) used colorimetry, and the remaining 18 (23%) facilities used an automated analyzer.

### Quality assurance

#### Guidelines and standard operating procedures (SOPs)

Out of the 122 facilities surveyed, 13 (11%) facilities reported to have the National Malaria Laboratory Diagnosis External Quality Assessment (EQA) Scheme Guideline [16]; two health facilities were observed using the protocol recommended in the guidelines; a QA protocol for malaria RDTs was available in nine (7%) facilities. SOPs for malaria microscopy and RDT were available in 12 (10%) and five (4%) facilities, respectively; job aids for microscopy were available in 30 (25%) facilities. Bench aids for malaria microscopy were available in 16 (13%) and displayed in 11 (69%) of these facilities; four facilities (3%) had displayed bench aids for RDTs.

#### Internal quality control and EQA

Among the 122 surveyed facilities, six (5%) facilities reported to have an internal quality control (QC) process, where slides were re-read by assigned staff within the laboratory; however, only one facility kept a record of internal QC data and two (2%) facilities were observed to archive malaria slides for EQA purposes.

Among the facilities surveyed 12 (10%) reported to be involved in EQA: three (2%) and nine (7.4%) facilities reported that external re-reading of slides was performed two to four times and once per year, respectively. The quality of smearing and staining of the malaria slides prepared in the assessed laboratories was observed and rated for 66 facilities: slides from only 11 (17%) facilities were found to be of excellent quality, with the remainder being of either good (31 (47%)) or poor (24 (36%)) quality (Table 6).

**Table 6:** Criteria for assessing quality of malaria blood film slide according to national malaria laboratory diagnosis external quality assessment scheme guideline

**Grade**	**Criteria for assessing the quality of malaria blood film smearing and staining characteristics**	**# facilities**
Excellent	**Gross appearance:** Both thin and thick film prepared on the same slide, thick film 10 mm diameter, newsprint read under thick film before staining, 10 mm from frosted end and thick film and between thick and a thin film with distinct head, body and tail.	11
	**Microscopic appearance:** Demonstrates RBCs lysed in thick film and a monolayer of RBCs, with normal and abnormal morphology in thin film. Staining allows the trophozoites, gametocytes and/or schizonts and the white blood cells to be clearly distinguished against the background.	
Good	**Gross appearance:** Film with uneven tail, too thick, too wide or too long with uneven thickness.	31
	**Microscopic appearance:** Demonstrates a monolayer of RBCs, and fixed RBCs. Staining allows the trophozoits, gametocytes and/or schizonts malaria parasites and the white blood cells to be clearly distinguished against the background.	
Poor	**Gross appearance:** Film with ragged tail, too thick, too wide or too long with uneven thickness.	24
	**Microscopic appearance:** Distorted appearance of the RBCs, malaria parasite and the white cells. Difficult to spot fields with monolayer of cells and distorted appearance of the RBCs, malaria parasite and the white cells.	

#### Supportive supervision

Supportive supervision had been provided within six months prior to the survey in 32 (26%) facilities. External bodies that provided the supervision included the Ministry of Health, the Ethiopian Public Health Institute (EPHI), ORHB, zonal health departments, and non-governmental organizations (NGOs).

#### Malaria case management

A test for malaria (microscopy or RDTs) was requested based on symptoms of patients in all the facilities surveyed. Clinical staff evaluating patients discussed the test results with laboratory personnel in 79 (65%) facilities. When clinicians disagreed with the laboratory test results, they independently made their own treatment decisions or discussed with the laboratory personnel the subsequent steps for diagnosis and patient management. Clinical personnel from 53 (51%) of the surveyed health facilities responded that they sometimes clinically treated patients with anti-malarial drugs while ignoring the laboratory test results, 28 (27%) responded that they order a repeat blood film to be made, and 16 (15%) responded that they further assess patients for other acute febrile illnesses. Very few clinicians responded that they used a combination of options, including clinical treatment and ordering RDT-testing (four (4%)), both clinical treatment and ordering a repeat blood film (two (2%)), or having the patient re-tested by microscopy as well as RDT (one (1%)).

## Discussion

Strong national health laboratory capacity is crucial to prevent, manage and ultimately control diseases such as malaria. In Ethiopia, 4,782,064 (47%) of the 10,088,744 total cases of suspected malaria were diagnosed using microscopy at the health centres or hospitals in 2012 [15]. The findings of the survey reported here confirm major findings from a previous, smaller assessment establishing a laboratory capacity-strengthening programme in Oromia. Although malaria laboratory diagnostic services were available in all facilities surveyed, significant gaps were observed in laboratory infrastructure, equipment, materials and reagent supply chains, human resource capacity, availability of policies, strategic plans and operational procedures, and laboratory services and data recording. This limitation clearly could be one of the main explanations as to why proportion of suspected malaria cases in Ethiopia remain unconfirmed and are treated presumptively.

Availability of water and electricity in assessed laboratories was high (>70%), but laboratory services were reportedly undermined by the frequent interruption of power and water, particularly if a back-up solution (e g, electric generators) was not available. Similarly to the assessment in 2009, a number of laboratories was observed to have gaps in equipment, materials and reagents (Tables 1, 2 nd 3), and some facilities were not able to provide certain services at all or, in the case of shortages, during certain times of the year. Thus, even though the national policy [3] states that microscopy should be used at health centre and hospital levels, more than half of surveyed facilities used RDTs, partially because functional microscopes were not available.

All laboratory personnel had at least diploma level training (i. e., two years). Pre-service training tends to focus on the technical theory of general laboratory skills (e. g., types of diagnostic tests, including haematology, chemistry) rather than developing practical malaria laboratory diagnosis skills (e. g., slide reading, species identification and parasite counting). While in-service training tends to be more practical, there is limited opportunity for in-service training; only a few facilities, had at least one staff participating in malaria microscopy refresher training during the previous year, quarter of the staff were provided with supportive supervision, and 12 (10%) were included in an EQA that involved external re-reading of slides. Although in-service training cannot replace comprehensive pre-service training, a well-thought-out, in-service training curriculum can significantly improve blood slide preparation and accuracy of microscopy [17]. Laboratory test requests are made for suspected malaria cases in all of the 122 health facilities, but against national policy ACT and chloroquine were still prescribed in a significant number of health facilities for suspected patients testing negative. Similar behaviour has been reported in numerous other malaria-endemic countries, including Tanzania and Zambia [18-21], which clearly undermines malaria prevention, case management and control efforts, as well as being medical malpractice (e. g., delaying treatment of infections of non-malarial aetiology could potentially result in deaths). Importantly, several studies have shown that withholding anti-malarial treatment of RDT-negative patients does not result in increased morbidity and mortality [22,23]. Generally, the findings indicate that clinicians do not rely on laboratory test results, because of malfunctioning and poorly maintained equipment [24], limited training of laboratory personnel, poor laboratory QA [19], lack of competency in clinical training or practice for malaria case management as shown here and elsewhere [25,26].

Only a few health facilities had SOPs, guidelines, bench and job aids for reference purposes. Coupled with the abovementioned challenges in infrastructure, equipment, reagents, supervision, and training, it was not surprising that a large proportion of health facilities were ranked as either good or poor when evaluated through the quality of prepared malaria microscopy slides.

Going forward, the malaria laboratory diagnosis and QA programme will focus on addressing needs and gaps identified that can be easily addressed, including supply of equipment (e. g., microscopes), reagents and materials (e. g., gloves, stains, slides), human resource capacity (e. g., through in-service training of laboratory and clinical staff, and supportive supervision), and availability of guidelines and job aids. Additionally, collaboration with EPHI and ORHB to implement an EQA scheme, which is currently being rolled out across Ethiopia, and availability and use of the updated National Malaria Case Management Guidelines at all levels in the health system will be strengthened [4]. Other needs and gaps, including rehabilitation of facilities, provision of continuous water and electricity, and provision of laboratory personnel will need additional financial investment. Together with the continued roll-out of multispecies RDTs at community level, it is hoped that increasing the laboratory capacity of health facilities through QA programmes [27] will ensure that fever cases in Ethiopia are increasingly accurately diagnosed. This should increase health service delivery overall, access of facilities by patients, as well as decrease the total cost of diagnosis and treatment (potentially by up to 46%) [28].

It could be considered that a possible caveat of the study is its design, i.e. a purposefully sampled number of facilities. However, given the number of facilities surveyed over an extensive geographic area, the findings’ likeness to a smaller, earlier study, as well as the study team’s knowledge of the context and infrastructure, the authors do not believe that the study’s outcome would have been different if another random selection of facilities would have been surveyed. Moreover, while they acknowledge that respondent bias may be questioned, the tool included a checklist that directed the study team to directly observe much of the facilities’ infrastructure, services and practices.

## Conclusions

The large investment in HIV/AIDS, tuberculosis and malaria has resulted in a dramatic scale-up of disease prevention (e g, long-lasting insecticide-treated nets for malaria) and curative services to remote populations. Unfortunately, laboratory capacity and services have not always kept pace with this expansion. The current standardized assessment provided a snapshot of laboratory capacity across sampled facilities in Ethiopia, showing significant gaps in supplies and equipment, as well as quality assurance and supportive supervision for malaria diagnosis. While baselines such as these are important in order to monitor and evaluate the impact of operational laboratory strengthening programming over time, they also show the multi-faceted challenges that such programming need to address in order to ensure that patients are correctly diagnosed and ultimately treated.

## Competing interests

The authors declare that they have no competing interests.

## Authors’ contributions

TA coordinated the assessment of health facilities, data entry and analysis, report writing and reviewed the manuscript. BH and YT were involved in coordinating the assessment, site selection, data entry, analysis, report writing and reviewed the manuscript. SG and AT were involved in reviewing the protocol, site selection, data collection and reviewed the manuscript. LD, FZ, MH, MT, DY, DT, AM, KG, and HB were involved in site selection, data collection and reviewed the manuscript. ZM and RR reviewed the study protocol and the manuscript. HT was involved in analysis and interpretation of the results and wrote the manuscript. All authors read and approved the final manuscript.

## References

[B1] WHO World Malaria Report 2013 Geneva, Switzerland: World Health Organization

[B2] Federal Ministry of Health National Strategic Plan for Malaria Prevention, Controland Elimination in Ethiopia 2010–2015 2009 Addis Ababa

[B3] Federal Ministry of Health Policy Planning Directorate Health and Health Related Indicators 2011 2013 Addis Ababa: Branna Press

[B4] Federal Ministry of Health National Malaria Guidelines 2011 Second Addis Ababa

[B5] Petti CA Polage CR Quinn TC Ronald AR Sande MA Laboratory medicine in Africa: a barrier to effective health care Clin Infect Dis 2006 42 377 382 10.1086/499363 16392084

[B6] Birx D De Souza M Nkengasong JN Laboratory challenges in the scaling-up of HIV, TB, and malaria programs: the interaction of health and laboratory systems, clinical research and service delivery Am J Clin Pathol 2009 131 849 851 10.1309/AJCPGH89QDSWFONS 19461092

[B7] Tynan A Atkinson JO Toaliu H Taleo G Fitzgerald L Whittaker M Riley I Schubert M Vallely A Community participation for malaria elimination in Tafea Province. Vanuatu: Part II. Social and cultural aspects of treatment-seeking behaviour Malar J 2011 10 204 10.1186/1475-2875-10-204 21787434 PMC3160431

[B8] World Health Organization Guidelines for the Treatment of Malaria 2010 Second Geneva

[B9] Federal Ministry of Health Annual Review Meeting Report 2011 Addis Ababa

[B10] National Bureau of Statistics Tanzania and Macro International Inc Tanzania Service Provision Assessment Survey 2006 2007 Dar-es-Salaam: National Bureau of Statistics and Macro International Inc

[B11] National Institute of Statistics (NIS) [Rwanda], Ministry of Health (MOH) [Rwanda], and Macro International Inc Rwanda Service Provision Assessment Survey 2007 2008 Calverton, Maryland, U.S.A: NIS, MOH, and Macro International Inc

[B12] Ministry of Health (MOH) [Uganda] and Macro International Inc Uganda Service Provision Assessment Survey 2007 2008 Kampala, Uganda: Ministry of Health and Macro International Inc

[B13] Kyabayinze DJ Achan J Nakanjako D Mpeka B Mawejje H Mugizi R Kalyango JN D’Alessandro U Talisuna A Van Geertruyden JP Parasite-based malaria diagnosis: are health systems in Uganda equipped enough to implement the policy? BMC Public Health 2012 12 695 10.1186/1471-2458-12-695 22920954 PMC3490993

[B14] Hailegiorgis B Girma S Melaku Z Teshi T Demeke L Gebresellasie S Yadeta D Tibesso G Whitehurst N Yamo E Carter J Reithinger R Laboratory malaria diagnostic capacity in health facilities in five administrative zones of Oromia Regional State, Ethiopia Trop Med Int Health 2010 15 1449 1457 10.1111/j.1365-3156.2010.02646.x 21040254

[B15] Federal Ministry of Health Malaria Commodity Micro-plan 2012 Unpublished Report 2013 Addis Ababa

[B16] Federal Ministry of Health Malaria Laboratory Diagnosis External Quality Assessment Scheme Guidelines 2009 Addis Ababa

[B17] Kiggundu M Nsobya S Kamya MR Filler S Nasr S Sorsey G Yeka A Evaluation of a comprehensive refresher training program in malaria microscopy covering four districts of Uganda Am J Trop Med Hyg 2011 84 820 824 10.4269/ajtmh.2011.10-0597 21540396 PMC3083754

[B18] Reyburn H Mbakilwa H Mwangi R Mwerinde O Olomi R Drakeley C Whitty CJM Rapid diagnostic tests compared with malaria microscopy for guiding outpatient treatment of febrile illness in Tanzania: randomised trial BMJ 2007 334 403 10.1136/bmj.39073.496829.AE 17259188 PMC1804187

[B19] Derua YA Ishengoma DRS Rwegoshora RT Tenu F Massaga JJ Mboera LEG Magesa SM Users’ and health service providers’ perception on quality of laboratory malaria diagnosis in Tanzania Malar J 2011 10 78 10.1186/1475-2875-10-78 21470427 PMC3084175

[B20] Hamer DH Ndhlovu M Zurovac D Fox M Yeboah-Antwi K Chanda P Sipilinyambe N Simon JL Snow RW Improved diagnostic testing and malaria treatment practices in Zambia JAMA 2007 297 2227 2231 10.1001/jama.297.20.2227 17519412 PMC2674546

[B21] Barat L Chipipa J Kolczak M Sukwa T Does the availability of blood slide microscopy for malaria at health centers improve the management of persons with fever in Zambia? Am J Trop Med Hyg 1999 60 1024 1030 10403337 10.4269/ajtmh.1999.60.1024

[B22] d’Acremont V Malila A Swai N Tillya R Kahama-Maro J Lengeler C Genton B Withholding antimalarials in febrile children who have a negative result for a rapid diagnostic test Clin Infect Dis 2010 51 506 511 10.1086/655688 20642354

[B23] Mubi M Janson A Warsame M Mårtensson A Källander K Petzold MG Ngasala B Maganga G Gustafsson LL Massele A Tomson G Premji Z Björkman A Malaria rapid testing by community health workers is effective and safe for targeting malaria treatment: randomised cross-over trial in Tanzania PLoS One 2011 6 e19753 10.1371/journal.pone.0019753 21750697 PMC3130036

[B24] Fonjungo PN Kebede Y Messele T Ayana G Tibesso G Abebe A Nkengasong JN Kenyon T Laboratory equipment maintenance: a critical bottleneck for strengthening health systems in sub-Saharan Africa? J Public Health Policy 2012 33 34 45 10.1057/jphp.2011.57 22071568

[B25] Polage CR Bedu-Addo G Owusu-Ofori A Frimpong E Lloyd W Zurcher E Hale D Petti CA Laboratory use in Ghana: physician perception and practice Am J Trop Med Hyg 2006 75 526 531 16968935

[B26] Chandler CI Jones C Boniface G Juma K Reyburn H Whitty CJ Guidelines and mindlines: why do clinical staff over-diagnose malaria in Tanzania? A qualitative study Malar J 2008 7 53 10.1186/1475-2875-7-53 18384669 PMC2323020

[B27] Marinucci F Manyazewal T Paterniti AD Medina-Moreno S Wattleworth M Hagembe J Redfield RR Impact of horizontal approach in vertical program: continuous quality improvement of malaria and tuberculosis diagnostic services at primary-level medical laboratories in the context of HIV care and treatment program in Ethiopia Am J Trop Med Hyg 2013 88 547 551 10.4269/ajtmh.12-0628 23324221 PMC3592539

[B28] Osei-Kwakye K Asante KP Mahama E Apanga S Owusu R Kwara E Adjei G Abokyi L Yeetey E Dosoo DK Punguyire D Owusu-Agyei S The benefits or otherwise of managing malaria cases with or without laboratory diagnosis: the experience in a district hospital in Ghana PLoS One 2013 8 e58107 10.1371/journal.pone.0058107 23505457 PMC3591456

